# Early sepsis recognition: a pilot study using a rapid high-multiplex host-response mRNA diagnostic test

**DOI:** 10.1186/s40635-025-00735-x

**Published:** 2025-02-21

**Authors:** Jingyi Lu, Michèle A. ter Voert, Mehtap Ünal, Natalie N. Whitfield, Oliver Liesenfeld, Jan C. ter Maaten, Timothy E. Sweeney, Hjalmar R. Bouma

**Affiliations:** 1 Department of Internal Medicine, University Medical Center Groningen, University of Groningen, P.O. Box 30.001, 9700 RB Groningen, The Netherlands; 2 Department of Acute Care, University Medical Center Groningen, University of Groningen, P.O. Box 30.001, 9700 RB Groningen, The Netherlands; 3 Inflammatix, Inc, Sunnyvale, CA 94085 USA; 4 Department of Clinical Pharmacy & Pharmacology, University Medical Center Groningen, University of Groningen, P.O. Box 30.001, 9700 RB Groningen, The Netherlands

**Keywords:** Acutelines, Sepsis, TriVerity, mRNA, Diagnosis, Prognosis, Host response, Acute infection

## Abstract

**Background:**

Early sepsis diagnosis is essential to allow timely initiation of adequate care. The TriVerity™ Test, performed on the Myrna™ Instrument, is the first rapid high-multiplex host-response mRNA diagnostic test that supports clinical decision-making by evaluating the likelihood of bacterial and/or viral infections and severity of illness. We present findings of the first, proof of concept, real-world evaluation in an emergency department (ED).

**Methods:**

Blood was collected in PAXgene^®^ Blood RNA tubes from adult patients visiting the ED with suspicion of infection between 4th December 2023 and 22nd January 2024. TriVerity was performed within 1 h (RNA extraction and amplification of 29 host mRNAs using LAMP technology on the Myrna Instrument within approximately 30 min). TriVerity generates three diagnostic scores (likelihood of bacterial infection, viral infection, and illness severity), each classified into five discrete interpretation bands (very low, low, moderate, high, and very high). Post hoc chart reviews were performed after hospital discharge to clinically adjudicate the infection status (consensus and forced adjudication).

**Results:**

Among 60 patients, there were 20 (33%) bacterial infections, 15 (25%) viral infections, 11 (18%) bacterial–viral coinfections and 14 (23%) did not have an infection under forced adjudication. Under consensus adjudication, bacterial results demonstrated 95% rule-in specificity and 95% rule-out sensitivity. Viral results demonstrated rule-in specificity 100% and 92% rule-out sensitivity. Since only three patients were admitted to the ICU or died in this cohort, we cannot draw firm conclusions about the predictive value of the test for severe endpoints.

**Conclusions:**

TriVerity is a rapid whole-blood host-response test to reliably detect the presence or absence of bacterial and/or viral infections, as well as to assess illness severity in patients presenting to the ED. Its quick turnaround time aligns with the ED workflow, offering timely insights for clinical decision-making. Results from upcoming large-scale validation studies will provide more detailed results on the diagnostic and prognostic accuracy of the test.

**Supplementary Information:**

The online version contains supplementary material available at 10.1186/s40635-025-00735-x.

## Background

Sepsis is a life-threatening dysregulated host response to infection, leading to organ dysfunction [1]. Although people of all ages can develop sepsis, newborns, the elderly, and people with comorbidities are at increased risk. The global burden is astonishingly high, with 20% of all deaths worldwide attributed to sepsis, affecting nearly 50 million people annually [2]. General practitioners (GPs) and emergency medical responders play a crucial role in pre-hospital recognition of sepsis in the Netherlands, yet their response often relies on 'gut feeling' due to the lack of objective, accurate diagnostic tests [3–5]. Following examination in the emergency department (ED), approximately one in four sepsis patients is admitted to the intensive care unit (ICU), making sepsis one of the most common reasons for ICU admission [6]. The in-hospital mortality rate is around 20% [7–9].

Despite the relatively high burden of sepsis, early recognition and prediction of its clinical course remain challenging due to the nonspecific and variable nature of its signs and symptoms. Current diagnostic criteria, such as the qSOFA score, have limited sensitivity (47%) for predicting sepsis in hospitalized patients [10]. While prompt initiation of antibiotic therapy and supportive care is crucial and can significantly lower mortality [8, 11–13], clinical deterioration still occurs in about 25% of sepsis patients within 24 h of hospital admission [14–16].

The TriVerity™ Test, performed on the Myrna™ Instrument, aims to address these challenges. TriVerity is an assay that generates three scores indicating the likelihood of bacterial infection, viral infection and severity of illness, based on the analysis of a combination of 29 mRNAs in whole blood measured via qRT-LAMP [17]. This first rapid high-multiplex host-response mRNA diagnostic test aids clinical decision-making in emergency departments by evaluating the likelihood of bacterial and/or viral infections and the severity of illness.

This study aims to evaluate the accuracy of TriVerity among patients with a suspected infection at the ED, marking the first real-world evaluation of the TriVerity Test system, supported by Acutelines' research infrastructure.

## Methods

### Study design and participants

Adult non-trauma patients (≥ 18 years of age) who presented at the ED of the University Medical Center Groningen (UMCG) between December 4, 2023, and January 22, 2024, with a suspected infection (as determined by the treating physician upon initial contact based on focal symptoms suggestive of an infection (e.g., productive cough, dyspnea, dysuria, pollakiuria, abdominal pain, erythema) and/or fever (≥ 38 °C, either at home or upon triage at the ED) and presented for internal medicine (i.e., general medicine, immunology, oncology, vascular medicine, hematology, nephrology, infectiology), gastroenterology, urology, non-trauma emergency medicine, pulmonology, or rheumatology. Patients who were transferred to our ED from another hospital after the initiation of therapy were excluded from the study.

Data and samples were prospectively collected by Acutelines, a data, image, and biobank at the emergency department of the University Medical Center Groningen (UMCG). To allow collection of data and biomaterials when applicable, primary screening of patients for eligibility is performed upon arrival in the ED by the ED-nurse together with a trained research team. For this study, blood samples were drawn into PAXgene^®^ Blood RNA tubes at triage, prior to treatment initiation, from patients presenting to the ED between 11:00 and 20:00 h. Patients were then examined, diagnosed, and treated according to the standard of care.

A deferred consent procedure (by proxy) is in place to allow the collection of data and biomaterials prior to obtaining written consent. If the patient or proxy could not reasonably be reached, an opt-out procedure was followed. A detailed description of the design, inclusion and exclusion criteria of Acutelines has been published previously [18].

### TriVerity test

The investigational use only (IUO) TriVerity Test (Inflammatix, Sunnyvale, CA, USA), performed on the IUO Myrna Instrument (Inflammatix, Sunnyvale, CA, USA), was performed on blood samples (2.5 mL) collected in PAXgene Blood RNA tubes within one hour of collection. The tubes were gently inverted 10 times to ensure thorough mixing of the PAXgene medium with the blood promoting lysis and RNA preservation, while avoiding foam formation. Following mixing, the samples were processed using a new TriVerity test cartridge and the Myrna instrument for subsequent testing.

**Fig. 1:**
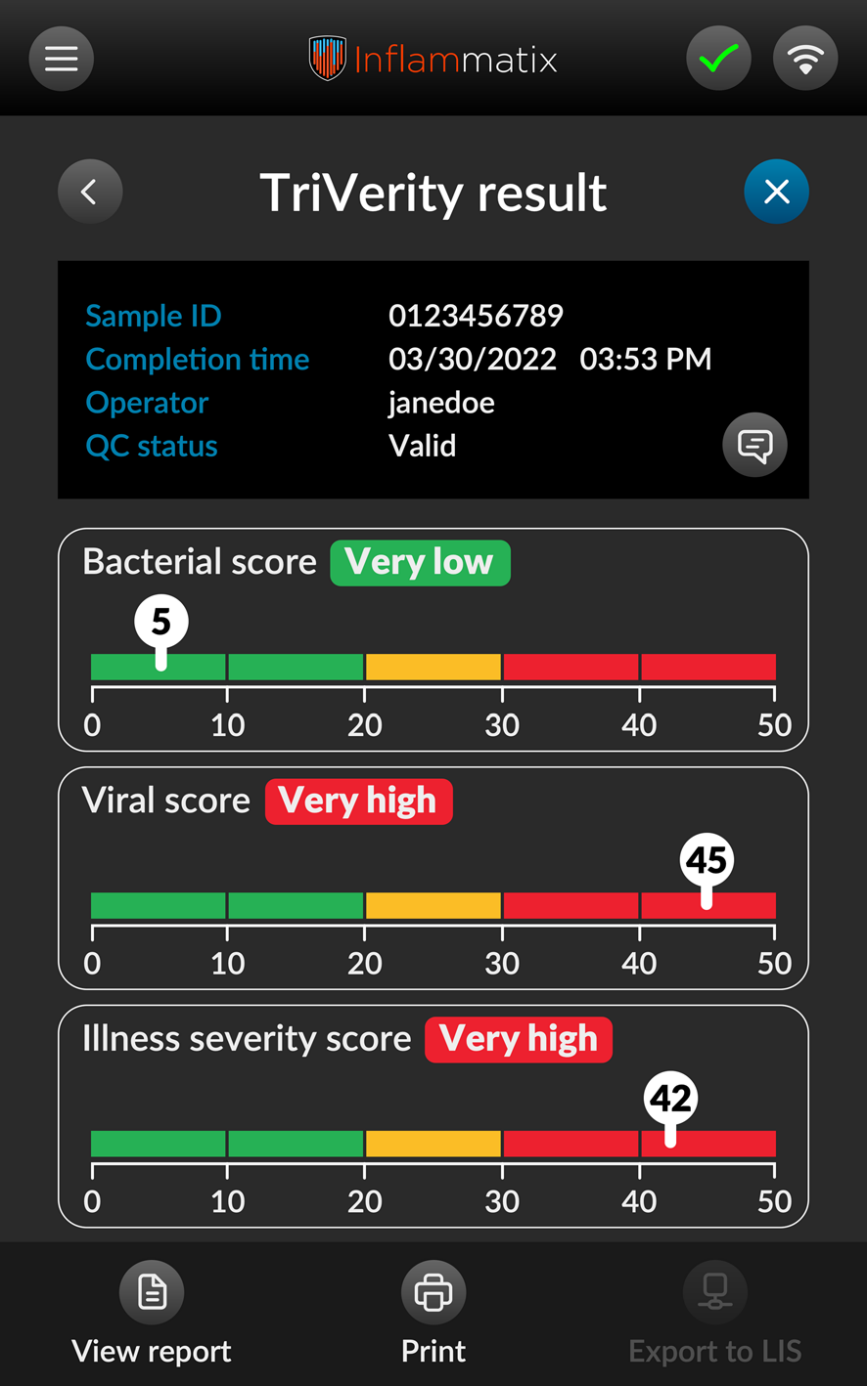
TriVerity results output image. The TriVerity Test System is not for sale. It is currently pending FDA clearance and has not received marketing approval or clearance from regulatory authorities in any jurisdiction

After approximately 30 min, The TriVerity Test, which involves RNA extraction, host gene amplification [19] and machine learning classifiers reading the gene expression, generates three distinct scores in approximately 30 min: likelihood of bacterial infection, viral infection, and illness severity **(**Fig. 1**).** These scores are presented in five discrete interpretation bands—very low, low, moderate, high, and very high. Each band offers an indication of the probability and severity of the infection, which may assist clinicians in making informed decisions about patient management.

### Data collection and processing

Patient data, including demographics, use of antibiotics and immunosuppressive drugs, and comorbidities were collected at baseline. Bedside monitoring data (i.e., vital parameters) were automatically captured and stored, and information from other data sources (such as the electronic health records) was securely imported. Study data were collected and managed using REDCap electronic data capture tools [20], hosted at the UMCG. The complete Acutelines protocol and a full data dictionary overview are available at www.acutelines.nl.

### Clinical adjudication of infection and severity endpoints

Post hoc chart reviews were conducted by two independent physicians to determine the adjudicated status of bacterial and viral infections for each case. Each case was assigned to one of four predefined categories—ruled out, unlikely, probable, or rule in—separately for bacterial and viral infections [21]. The detailed definition of the adjudication categories can be found in **Supplemental **Table 1. In instances where the two adjudicators disagreed, a third reviewer made the final determination. All adjudicators were blinded to the TriVerity bacterial and viral results.

**Table 1:** Patient demographics segmented by adjudicated infection status established using forced adjudication

Variable	All patients	No infection	Only bacterial infection	Only viral infection	Bacterial and viral infection	*p*
	*n* = 60	*n* = 14	*n* = 20	*n* = 15	*n* = 11	
Baseline characteristics						
Age, yr, median [IQR]	66.00 [54.75, 75.00]	74.00 [49.00, 76.75]	60.50 [51.75, 75.00]	68.00 [64.00, 76.00]	63.00 [55.50, 68.00]	0.450
Sex, male, n (%)	34 (56.7)	8 (57.1)	11 ( 55.0)	10 ( 66.7)	5 (45.5)	0.754
Sepsis, n (%)	7 (11.7)	0 (0.0)	5 ( 25.0)	0 ( 0.0)	2 (18.2)	0.052
Hospital admission, n (%)	32 (53.3)	5 (35.7)	13 ( 65.0)	7 ( 46.7)	7 (63.6)	0.311
Use of immunocompromised drug, n (%)	14 (23.3)	1 (7.1)	4 ( 20.0)	6 ( 40.0)	3 (27.3)	0.204
Previous antibiotics, n (%)	21 (35.0)	3 (21.4)	6 ( 30.0)	7 ( 46.7)	5 (45.5)	0.427
Charlson Comorbidity Index, median [IQR]	3.00 [2.00, 5.25]	3.00 [2.00, 6.00]	3.00 [2.00, 6.00]	5.00 [2.50, 5.00]	3.00 [1.00, 4.50]	0.745
Vital signs at presentation						
Hear rate, bpm, median [IQR]	96.00 [84.25, 113.00]	97.50 [73.50, 111.25]	97.50 [89.75, 117.25]	93.00 [78.50, 101.00]	93.00 [90.50, 109.00]	0.284
SBP, mmHg, median [IQR]	137.50 [123.00, 150.50]	138.50 [124.25, 169.25]	131.00 [121.50, 150.50]	141.00 [132.00, 147.50]	137.00 [127.50, 146.00]	0.713
DBP, mmHg, median [IQR]	80.50 [73.00, 89.25]	84.00 [80.00, 85.75]	88.00 [72.50, 94.25]	75.00 [73.00, 84.50]	73.00 [70.00, 83.50]	0.257
Temperature, °C, median [IQR]	37.10 [36.68, 37.73]	36.75 [36.28, 37.20]	37.55 [36.80, 38.15]	37.40 [36.70, 37.95]	37.10 [36.75, 37.45]	0.040
Respiratory frequency, breaths per minute, median [IQR]	20.00 [17.00, 23.00]	20.00 [19.00, 23.00]	18.00 [16.00, 23.25]	20.00 [16.00, 23.00]	19.50 [18.00, 22.50]	0.741
Glasgow Coma Scale, median [IQR]	15.00 [15.00, 15.00]	15.00 [15.00, 15.00]	15.00 [15.00, 15.00]	15.00 [15.00, 15.00]	15.00 [15.00, 15.00]	0.239
qSOFA, median [IQR]	0.00 [0.00, 1.00]	0.00 [0.00, 1.00]	0.00 [0.00, 1.00]	0.00 [0.00, 1.00]	0.00 [0.00, 0.50]	0.838
SIRS, median [IQR]	2.00 [1.00, 2.00]	1.00 [1.00, 2.00]	2.00 [1.00, 3.00]	1.00 [1.00, 2.00]	2.00 [1.00, 2.00]	0.287
Biomarkers						
WBC, 10e9 cells/L, median [IQR]	9.80 [7.35, 13.43]	8.95 [6.68, 11.17]	12.95 [9.78, 14.95]	7.50 [4.95, 10.45]	12.00 [6.45, 13.75]	0.005
CRP, mg/L, median [IQR]	69.50 [13.00, 130.25]	10.50 [7.42, 13.75]	104.50 [61.00, 179.25]	35.00 [8.50, 79.50]	149.00 [93.00, 233.00]	< 0.001
Lactate, mmol/L, median [IQR]	1.30 [1.00, 1.80]	1.60 [1.10, 2.00]	1.70 [1.00, 1.90]	1.30 [1.10, 1.70]	1.05 [1.00, 1.43]	0.438
Outcome						
7-day ICU admission or mortality, n (%)	3 ( 5.0)	0 ( 0.0)	1 (5.0)	1 (6.7)	1 (9.1)	0.750

Continuous variables are expressed as median with interquartile range (IQR). Categorical variables are represented as proportions. A p-value of < 0.05 was considered statistically significant

SBP, systolic blood pressure; DBP, diastolic blood pressure; qSOFA, Quick Sequential Organ Failure Assessment; SIRS, systemic inflammatory response syndrome; WBC, white blood cell count; CRP, C-reactive protein; IQR = interquartile range

The dataset was used in two ways to convert the four original adjudication categories into a single binary classification for each infection type (i.e., bacterial or viral infection present or absent). To create a unified 'ground truth' label with three possible outcomes (bacterial infection, viral infection, or non-infected), we applied two distinct classifications to the data: 'consensus adjudication' (CA) and 'forced adjudication' (FA).

In the CA approach, only cases classified as 'Rule In' were deemed positive for bacterial or viral infection, while cases marked as 'Ruled Out' were considered absent of the infection. Cases classified as 'Unlikely' or 'Probable' were treated as inconclusive and excluded from further analysis. In contrast, the FA approach required that all cases be classified into binary categories. Here, cases adjudicated as either 'Probable' or 'Rule In' were labeled as positive for bacterial or viral infection, while cases rated as 'Unlikely' or 'Ruled Out' were considered absent of the infection. This provided a broader interpretation to allow analysis of all cases in the dataset.

The primary endpoint of severity was defined as ICU admission and/or mortality due to infection within 7 days of ED presentation. In addition to the patients that were admitted to the ICU or died of an infection, two patients were admitted to the ICU for a non-infectious reason (cardiac arrest secondary to hypoxemia following an allergic reaction to CT-contrast, and ICU admission because of shortage of high-flow oxygen bed at the regular ward) and one patient with advanced esophageal cancer died due to a perforated esophagus. These patients were not labeled as meeting the “severity endpoint”, which concerned only ICU admission or death due to an infection. Physicians were blinded to the TriVerity results.

### Statistical analysis

Continuous variables are expressed as median with interquartile range (IQR). Categorical variables are represented as proportions. A p-value of < 0.05 was considered statistically significant. The performance of TriVerity test was evaluated in two ways: [1] using TriVerity scores through the area under the receiver operating characteristics (AUROCs) and [2] using TriVerity interpretation bands to calculate sensitivity, specificity, likelihood ratios, and positive and negative predictive values [22]. The calculation basis of TriVerity bands performance is demonstrated in Supplemental Table 2. Statistical analyses and figures generation were conducted using R version 4.3.3.

**Table 2:** Performance of TriVerity results after applying previously established cutoffs to segment scores into clinically interpretable bands

Performance of TriVerity bacterial bands								
Test interpretation band	CA infection status		Sens	Spec	LR	PPV	NPV	% Patients in band
	Bacterial Inf. Present	Bacterial Inf Absent						
Very high	6	1	0.29	0.95	6.29	86%	58%	16%
High	5	0	0.24	1	Inf	100%	58%	12%
Moderate	7	8	0.33	0.64	0.92	47%	50%	35%
Low	2	5	0.9	0.23	0.42	53%	71%	16%
Very low	1	8	0.95	0.36	0.13	59%	89%	21%

Performance of TriVerity viral bands								
Test interpretation band	CA infection status		Sens	Spec	LR	PPV	NPV	% Patients in band
	Viral Inf Present	Viral Inf Absent						
Very high	11	0	0.44	1	Inf	100%	71%	19%
High	6	3	0.24	0.91	2.72	67%	62%	15%
Moderate	3	9	0.12	0.74	0.45	25%	53%	20%
Low	3	10	0.88	0.29	0.41	48%	77%	22%
Very low	2	12	0.92	0.35	0.23	51%	86%	24%

Performance of TriVerity illness severity bands					
Test interpretation band	Ground truth		Spec	NPV	% Patients in band
	7-day ICU admission or mortality	Survival and/or Discharge			
Very high	0	3	0.95	95%	5%
High	0	8	0.86	94%	13%
Moderate	2	11	0.81	98%	22%
Low	0	20	0.35	100%	33%
Very low	1	15	0.26	94%	27%

Results of bacterial and viral scores were reported based on adjudicated infection status under consensus adjudication (CA). Sens. = sensitivity, Spec. = specificity, LR = likelihood ratio, PPV = positive predictive value, NPV = negative predictive value

## Results

### Patient characteristics

**Fig. 2:**
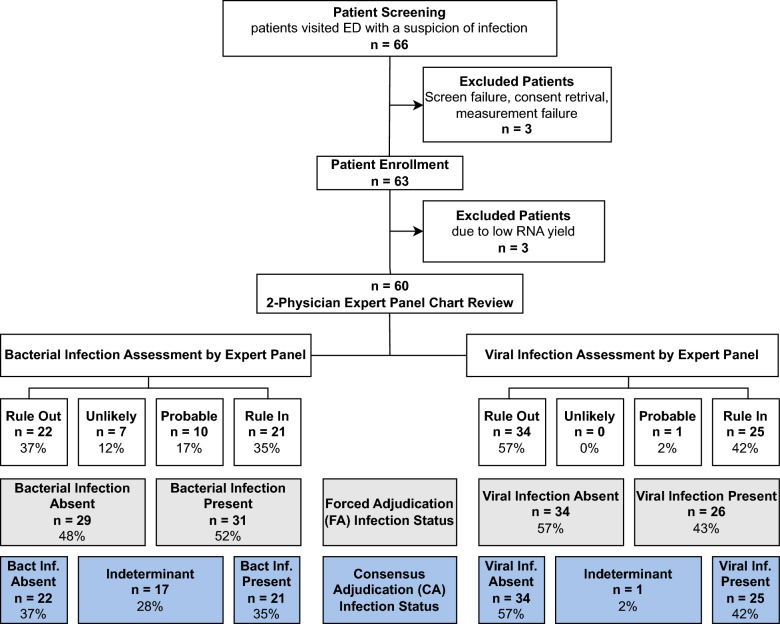
Study flowchart. To assess the diagnostic performance of TriVerity, we screened 66 patients presenting to the emergency department (ED) with a clinical suspicion of acute infection. After excluding 3 patients, our cohort with 63 patients had whole blood samples tested with TriVerity. TriVerity results from 3 patients were removed from analysis due to low RNA yield, leading to a final cohort of 60 patients. An expert panel of two physicians, blinded to TriVerity results, reviewed medical chart data to determine infection status. The physicians assessed each patient’s infection status on a 4-point scale (ruled out, unlikely, probable, and rule in). These assessments were then converted into binary categories of “present” or “absent” for bacterial and/or viral infection using two methods: a conservative consensus adjudication (CA) and a liberal forced adjudication (FA)

In this study, we enrolled 66 consecutive adult patients with suspected acute infection from December 2023 until February 2024. After excluding 3 patients due to low RNA yield and 3 due to withdrawal of consent, the final cohort comprised 60 patients (Fig. 2). The median age of the cohort was 66 years (IQR 55–75 years), with 57% being male. Results of the expert panel chart review assessments (with 81% inter-observer agreement) and subsequent classifications for the presence of bacterial and viral infections under FA and CA are summarized in Fig. 2.

Under FA, 20 (33%) had a bacterial infection, 15 (25%) had a viral infection, 11 (18%) had a coinfection (i.e., bacterial and viral), and 14 (23%) did not have an infection. Under CA, which excludes patients with inconclusive infections, 21 patients (35%) had a bacterial infection present, and 25 patients (42%) had a viral infection present.

Detailed clinical characteristics of the patient cohort segmented by FA are summarized in Table 1. The median heart rate (HR) was 96.0 bpm (IQR 84.2–113.0 bpm), the median systolic blood pressure (SBP) was 137.5 mmHg (IQR 123.0–150.5 mmHg), and the median diastolic blood pressure (DBP) was 80.5 mmHg (IQR 73.0–89.2 mmHg). The median body temperature was 37.1°C (IQR 36.7–37.7°C), and the median respiratory frequency (RF) was 20.0 breaths per minute (IQR 17.0–23.0 breaths per minute). The median C-reactive protein (CRP) level was 76.0 mg/L (IQR 20.0–131.0 mg/L), and the median lactate level was 1.40 mmol/L (IQR 1.20–1.72 mmol/L). In total, 2 patients had a Quick Sequential Organ Failure Assessment (qSOFA) score greater than 1, 12 patients met more than 2 criteria for Systemic Inflammatory Response Syndrome (SIRS). and 7 patients were classified as having sepsis according to diagnosis. The hospital admission rate for the cohort was 56.7%. Three patients reached the severity endpoint (ICU admission or deceased within 7 days after ED presentation), all of whom had pulmonary infections and required respiratory and/or circulatory support.

### Performance of TriVerity bacterial, viral and severity scores

**Fig. 3:**
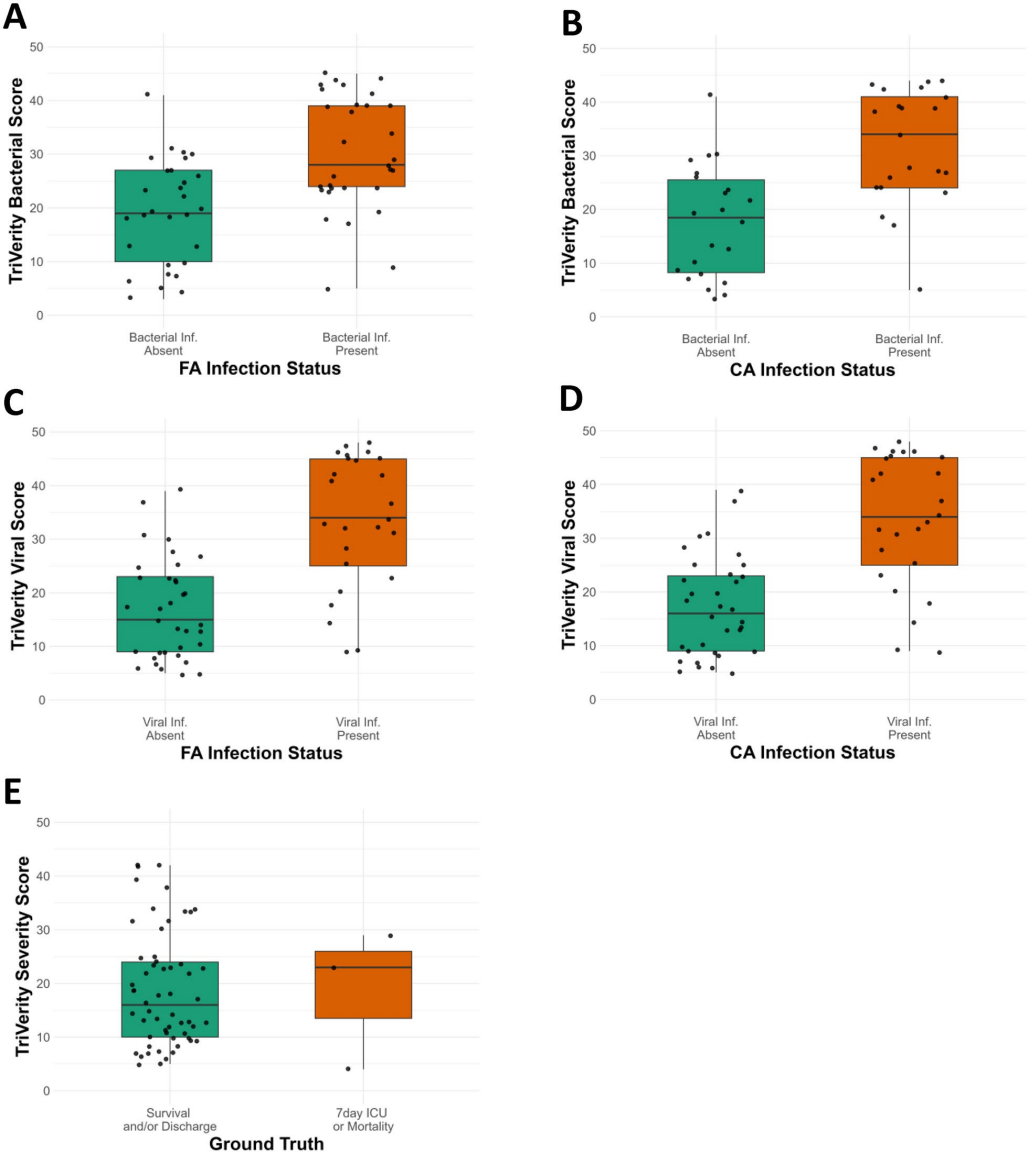
Distribution of TriVerity bacterial and viral scores stratified by infection statuses adjudicated through forced adjudication (FA) and consensus adjudication (CA), along with TriVerity severity scores categorized by patients with ICU admission or death within 7 days versus survival and/or discharge. A Distribution of bacterial scores by infection status under FA; B distribution of bacterial scores by infection status under CA; C distribution of viral scores by infection status under FA; D distribution of viral scores by infection status under CA; E distribution of severity scores by ICU admission or mortality within 7 days versus survival and/or discharge

The distribution of TriVerity bacterial scores and viral scores segmented by CA and FA adjudicated infection statuses is provided in Fig. 3. To assess the performance of TriVerity bacterial and viral scores, we calculated AUROCs for distinguishing the presence of bacterial or viral infection.

Under CA, the TriVerity bacterial score performed with an AUROC of 0.81 (95% CI, 0.68–0.94) for distinguishing patients with proven and ruled-out bacterial infection. Under FA, which included patients with uncertain infection status, the TriVerity bacterial score distinguished bacterial infection patients with an AUROC of 0.76 (95% CI, 0.64–0.88).

The TriVerity viral score performed with an AUROC of 0.85 (95% CI, 0.74–0.95) for distinguishing patients with proven versus ruled-out viral infection under CA. Under FA, TriVerity viral score demonstrated an AUROC of 0.85 (95% CI, 0.75–0.95).

In addition to supporting a bacterial or viral infection diagnosis, the prediction of severity outcomes using the TriVerity Test was also evaluated. Figure 3 shows the distribution of TriVerity severity scores segmented by ICU admission or death within 7 days vs. survival and/or discharge.

### TriVerity (actionable) interpretation bands

TriVerity incorporates five interpretation bands to allow for clinical actionability. Clinical actionability was defined as a rule-in or rule-out result for either bacterial or viral infection from a TriVerity test result. The performance of the bacterial and viral bands under CA and FA is shown in Table 2 and Supplemental Table 3. Specifically, the bacterial infection results demonstrated 95% specificity for ruling in bacterial infections and 95% sensitivity for ruling them out. For viral infections, the results showed 100% specificity for ruling in viral infections and up to 92% sensitivity for ruling them out.

Due to the small number of patients meeting the criteria for severe outcomes, the interpretation of the TriVerity illness severity score is limited to the cases involved in negative class. Among the 57 patients without severe outcomes, 60% received very low or low bands, with negative predictive value of 94% and 100%, respectively.

## Discussion

The rapid diagnosis of acute infections and the accurate prediction of disease severity in the ED remains a critical unmet need. In this study, we utilized TriVerity, a high-multiplex mRNA diagnostic test, to measure samples from patients presenting to the ED to determine the likelihood of bacterial or viral infections, as well as to predict the necessity for escalated medical care. We prospectively validated TriVerity using fresh blood samples collected from 60 patients presenting to the ED with suspected infections. Most patients were having bacterial and/or viral infections but were not critically ill. Our results demonstrated that TriVerity exhibited high accuracy in diagnosing both bacterial and viral infections. The performance of TriVerity in predicting severe outcomes requires further investigation in a larger sample size. Furthermore, the rapid turnaround time and streamlined TriVerity workflow supports its feasibility for routine clinical use.

While TriVerity demonstrated high accuracy in identifying bacterial and viral infections, a few cases showed discrepancies between TriVerity interpretation bands and clinical adjudication. Several factors may have contributed to the divergent results, including the initiation of potentially effective antibiotic therapy before ED presentation, which led to two false-negative bacterial infection cases; a viral infection with a high cycle threshold (Ct > 30), resulting in one false-negative viral case; and immunocompromised status in four patients. Although TriVerity accurately identified most non-severe cases, there were several instances where the test predicted a severe course, yet the patients were neither admitted to the ICU nor died within 7 days. However, retrospective chart reviews indicated that all patients with positive severe predictions were hospitalized. Of these, one patient presented with hemodynamic instability in the ED, four patients required significant escalation of care during hospitalization, and the majority was immunocompromised.

If the TriVerity result was in the moderate band, which was the case in 35% of bacterial infections, 20% of viral infections, and 22% of severity predictions, it is unlikely to influence clinical decision-making. While this holds true for individual scores (bacterial/viral/illness severity), the TriVerity three-score system provides both diagnostic and prognostic insights (Fig. 1). In this cohort, 85% of patients (51/60) received at least one result classified as very low, low, high, or very high, supporting clinical decision-making in most cases. Only three patients (5%) had moderate results across all axes. However, given the small sample size, this percentage may not be conclusive. A larger Sepsis-Shield trial with over 1,200 emergency department patients found that 99.6% of consensus-adjudicated cases fell into a clinically actionable category (very low, low, high, or very high) for at least one diagnostic or prognostic axis (infection or severity) [23].

Currently, there are no rapid diagnostic tools available that can reliably diagnose infection caused by both bacterial and viral pathogens. In the ED, clinical decisions regarding treatment for bacterial and/or viral infections are typically based on a combination of rapid viral respiratory tests, inflammatory markers, and the physician's clinical judgment [24–26]. Diagnostic results from cultures (e.g., blood, sputum, or feces) obtained during hospitalization may later modify the initial diagnosis and prompt treatment adjustments, but this process is time-consuming and can lead to overuse of diagnostic tests and antimicrobial therapies [27]. In predicting poor outcomes in patients with suspected infections, one commonly used tool is the qSOFA, a rapid bedside clinical score designed to assess the risk of in-hospital mortality and ICU stays exceeding three days [28]. The prognostic performance of qSOFA for predicting in-hospital mortality shows a sensitivity of 70% (95% CI, 59%-80%) and a specificity of 79% (76%-82%) [29], with similar results for predicting ICU admission.

Complementary to commonly used clinical scores like qSOFA and SIRS, which rely on vital signs at triage, TriVerity leverages split-well multiplexed RT-qLAMP and machine learning technology to analyze gene expression patterns in white blood cells [30]. By capturing the host immune response, TriVerity surpasses traditional blood-based diagnostics that only detect pathogens present in the bloodstream [31]. It enables the detection and prediction of immune responses to bacterial or viral pathogens, as well as non-pathogenic tissue damage [17, 32]. Moreover, gene expression changes reflect not only disease-induced molecular alterations, but also temporal heterogeneity, driven by the significant dynamic shifts as sepsis progresses through different phases [27]. Integrating results from a host response analysis, as can be performed by TriVerity, with currently used clinical scores used in sepsis screening such as qSOFA and/or SIRS could enhance predictive capabilities and improve clinical decision-making.

The ability to measure whole-blood gene expression profiles at the bedside with results available in approximately 30 min, offers significant potential for incorporating transcriptomics into clinical practice, supporting clinical trial enrichment and personalizing treatment decisions. Previous studies using microarray analysis in ICU patients and RNAseq in ED cohorts [33] have demonstrated the value of gene expression analysis in critically ill patients. Moreover, molecular diagnostic system based on gene expression profiling could predict responses to adjunctive or targeted therapies, thereby enriching patient populations in clinical trials (34). These insights could guide the use of targeted therapies and further enhance clinical trial outcomes.

## Limitations

Although this is the first proof-of-concept real-world study using the TriVerity Test system at the ED, the study is limited by its single-center design in a tertiary care center, which can limit generalizability to small rural hospitals. Nonetheless, this hospital has a substantial geographical spread in a rural area, ensuring a diverse population.

One potential limitation is the high proportion of immunocompromised patients in this center. As noted earlier, we observed false positive and false negative results in this group. The impact of immunosuppressive medications on gene expression levels, and consequently on TriVerity results, remains unclear. Larger studies are needed to determine whether TriVerity is effective in this subpopulation. Future research focused on immunocompromised patients and those receiving antibiotic therapy could further refine the performance of TriVerity. These studies could provide critical insights into optimizing the use of TriVerity results to support clinical decision-making in high-risk populations.

The study population is limited to adult medical patients admitted for internal medicine (including nephrology, hematology, oncology, general medicine, allergology, infectiology), rheumatology, gastroenterology, pulmonology, urology, and emergency medicine. Patients admitted to the emergency department for other specializations were not screened as the incidence of infections and sepsis is very low among these patients. For practical reasons, only patients visiting the ED between 11:00 – 20:00 h were included. Although we do not have evidence to support that this inclusion time may have caused selection bias, the risk of such bias should be taken into account when interpreting the evidence provided in the study.

Finally, another limitation is its small sample size, particularly among patients with severe outcomes. Yet, we do not consider the small sample size an issue when interpreting the proof-of-concept results about feasibility, and we decided to supply additional information about discrepancies between the TriVerity interpretation band and adjudication.

Further multi-center, large-scale validation studies are necessary to more comprehensively demonstrate the performance and reliability of the TriVerity Test system in diverse clinical settings; such a study has recently been completed and results are expected in due time.

## Conclusions

TriVerity is a rapid host-response test to reliably identify the presence or absence of bacterial and/or viral infections and the associated illness severity in patients visiting the ED. Additional analysis is needed to assess its prognostic performance to predict adverse outcomes. The interpretation bands of TriVerity may facilitate precise clinical decision-making, enabling healthcare providers to quickly and accurately identify patients with high or low probabilities of bacterial or viral infections, thereby optimizing treatment strategies and potentially improving patient outcomes.

## Supplementary Information

Supplementary material 1.

## Data Availability

The datasets used and/or analyzed during the current study are available from the corresponding author on reasonable request.
