# Supplementary material for: Early sepsis recognition: a pilot study using a rapid high-multiplex host-response mRNA diagnostic test
Source: Intensive Care Med Exp. 2025 Feb 21;13:21. doi: 10.1186/s40635-025-00735-x (PMC11845329; doi:10.1186/s40635-025-00735-x)
Supplement: Supplementary file 1 — Supplementary material 1. [file 40635_2025_735_MOESM1_ESM.docx]

**Supplemental Table 1.** Definition of Adjudication Categories

| Definition |  |
| --- | --- |
| 1 | **Ruled Out**: [Bacterial/Viral] infection ruled out by clinical assessment, with another definitive diagnosis established. |
| 2 | **Unlikely**: [Bacterial/Viral] infection unlikely based on clinical assessment, but no other definitive diagnosis established, or a microorganism detected but interpreted as colonization rather than a cause of infection. |
| 3 | **Probable**: [Bacterial/Viral] infection probable based on clinical assessment, but not confirmed by microbiological, PCR, or other laboratory results. |
| 4 | **Rule In**: [Bacterial/Viral] infection proven by clinical assessment, supported by microbiological or other laboratory test results. |

**Supplemental Table 2.** Calculation of accuracy for bacterial and viral interpretation bands.

| **Test interpretation band (band number)** | **Infection status by Clinical adjudication** | | **Sensitivity** | **Specificity** | **Likelihood ratio (LR)** |
| --- | --- | --- | --- | --- | --- |
| Very high (5) | yes ($y_{5}$) | no ($x_{5}$) | $\frac{y_{5}}{\sum_{n=1}^{5} y_{n}}$ | $\frac{x_{4}+x_{3}+x_{2}+x_{1}}{\sum_{n=1}^{5} x_{n}}$ | $\frac{sensitivity}{1-specificity}$ |
| High (4) | yes ($y_{4}$) | no ($x_{4}$) | $\frac{y_{4}}{\sum_{n=1}^{5} y_{n}}$ | $\frac{x_{5}+x_{3}+x_{2}+x_{1}}{\sum_{n=1}^{5} x_{n}}$ | $\frac{sensitivity}{1-specificity}$ |
| Moderate (3) | yes ($y_{3}$) | no ($x_{3}$) | $\frac{y_{3}}{\sum_{n=1}^{5} y_{n}}$ | $\frac{x_{1}+x_{2}+x_{4}+x_{5}}{\sum_{n=1}^{5} x_{n}}$ | $\frac{sensitivity}{1-specificity}$ |
| Low (2) | yes ($y_{1}$) | no ($x_{1}$) | $\frac{y_{5}+y_{4}+y_{3}+y_{1}}{\sum_{n=1}^{5} y_{n}}$ | $\frac{x_{2}}{\sum_{n=1}^{5} x_{n}}$ | $\frac{1-sensitivity}{specificity}$ |
| Very low (1) | yes ($y_{1}$) | no ($x_{1}$) | $\frac{y_{5}+y_{4}+y_{3}+y_{2}}{\sum_{n=1}^{5} y_{n}}$ | $\frac{x_{1}}{\sum_{n=1}^{5} x_{n}}$ | $\frac{1-sensitivity}{specificity}$ |

*Same calculation for severity with the respective endpoint.

**Supplemental Table 3.** Performance of TriVerity results after applying previously established cutoffs to segment scores into clinically interpretable bands.

| **Performance of TriVerity Bacterial Bands** | | | | | | | | | |
| --- | --- | --- | --- | --- | --- | --- | --- | --- | --- |
| Test interpretation band | FA Infection Status | | | Sens. | Spec. | LR | PPV | NPV | % Patients in Band |
|  | Bacterial Inf. Present | Bacterial Inf.  Absent | |  |  |  |  |  |  |
| **Very high** | 7 | 1 | | 0.23 | 0.97 | 6.55 | 88% | 54% | 13% |
| **High** | 7 | 1 | | 0.23 | 0.97 | 6.55 | 88% | 54% | 13% |
| **Moderate** | 12 | 11 | | 0.39 | 0.62 | 1.02 | 52% | 49% | 38% |
| **Low** | 3 | 8 | | 0.9 | 0.28 | 0.35 | 57% | 73% | 18% |
| **Very low** | 2 | 8 | | 0.94 | 0.28 | 0.23 | 58% | 80% | 17% |
| **Performance of TriVerity Viral Bands** | | | | | | | | | |
| Test interpretation band | FA Infection Status | | | Sens. | Spec. | LR | PPV | NPV | % Patients in Band |
|  | Viral Inf.  Present | | Viral Inf.  Absent |  |  |  |  |  |  |
| **Very high** | 11 | | 0 | 0.42 | 1 | Inf | 100% | 69% | 18% |
| **High** | 6 | | 3 | 0.23 | 0.91 | 2.62 | 67% | 61% | 15% |
| **Moderate** | 3 | | 9 | 0.12 | 0.74 | 0.44 | 25% | 52% | 20% |
| **Low** | 3 | | 10 | 0.88 | 0.29 | 0.39 | 49% | 77% | 22% |
| **Very low** | 3 | | 12 | 0.88 | 0.35 | 0.33 | 51% | 80% | 25% |

Results of bacterial and viral scores were reported based on adjudicated infection status under forced adjudication (FA). Sens. = sensitivity, Spec. = specificity, LR = likelihood ratio, PPV = positive predictive value, NPV = negative predictive value.
